# Breeding Wheat for Durable Leaf Rust Resistance in Southern Africa: Variability, Distribution, Current Control Strategies, Challenges and Future Prospects

**DOI:** 10.3389/fpls.2020.00549

**Published:** 2020-05-15

**Authors:** Sandiswa Figlan, Khayalethu Ntushelo, Learnmore Mwadzingeni, Tarekegn Terefe, Toi J. Tsilo, Hussein Shimelis

**Affiliations:** ^1^School of Agricultural, Earth and Environmental Sciences, University of KwaZulu-Natal, Pietermaritzburg, South Africa; ^2^Agricultural Research Council-Small Grain, Bethlehem, South Africa; ^3^Department of Agriculture and Animal Health, University of South Africa, Florida, South Africa

**Keywords:** durable resistance, pathogen variability, *Puccinia triticina*, southern Africa, virulence, wheat breeding

## Abstract

Leaf or brown rust of wheat caused by *Puccinia triticina* (*Pt*) is one of the most damaging diseases globally. Considerable progress has been made to control leaf rust through crop protection chemicals and host plant resistance breeding in southern Africa. However, frequent changes in the pathogen population still present a major challenge to achieve durable resistance. Disease surveillance and monitoring of the pathogen have revealed the occurrence of similar races across the region, justifying the need for concerted efforts by countries in southern Africa to develop and deploy more efficient and sustainable strategies to manage the disease. Understanding the genetic variability and composition of *Pt* is a pre-requisite for cultivar release with appropriate resistance gene combinations for sustainable disease management. This review highlights the variability and distribution of the *Pt* population, and the current control strategies, challenges and future prospects of breeding wheat varieties with durable leaf rust resistance in southern Africa. The importance of regular, collaborative and efficient surveillance of the pathogen and germplasm development across southern Africa is discussed, coupled with the potential of using modern breeding technologies to produce wheat cultivars with durable resistance.

## Introduction

The fungal disease, leaf rust caused by *Puccinia triticina* Eriks. (*Pt*), is widely distributed across all the major wheat-growing regions in the world (Saari and Prescott, 1985; Kolmer, 2013). It is considered as one of the most common diseases of bread wheat (*Triticum aestivum*), durum wheat (*Triticum turgidum* var. *durum*), and triticale (*X Triticosecale*; Bolton et al., 2008; Huerta-Espino et al., 2011). The pathogen is an obligate biotrophic fungus mainly infecting the leaves of wheat at various growth stages but can also infect the leaf sheath and glumes. The infection causes grain yield losses of more than 50% in susceptible cultivars (Hussein et al., 2005; Huerta-Espino et al., 2011; Terefe et al., 2011).

The southern African sub-region is the main epidemiological zone of leaf rust, exposing most wheat cultivars to infection and yield loss (Saari and Prescott, 1985; Huerta-Espino et al., 2011). Since the 1980s, several South African wheat cultivars have been reported to be susceptible to leaf rust, with localized epidemics frequently occurring in the winter rainfall regions of the Western Cape Province and irrigated areas in other provinces (Pretorius et al., 2007; Terefe et al., 2009). Recently, low infection levels have been reported in farmers’ fields due to low inoculum levels, mainly resulting from repeated fungicide applications (Terefe et al., 2009). However, breeding for host plant resistance which is a pre-emptive, effective and a more sustainable way to control future leaf rust epidemics, should be the main objective of wheat breeding programs in southern Africa. This will reduce heavy reliance on fungicide applications which could have a negative impact on the environment.

Collections of *Pt* field isolates from countries in southern Africa including Malawi, South Africa, Zambia, and Zimbabwe, resulted in the identification of more than 20 physiologic races to date (Pretorius et al., 1987, 1990, 2015; Pretorius and Le Roux, 1988; Terefe et al., 2011; Boshoff et al., 2018). Analysis of the collected isolates revealed genetic, and virulence similarities among the major races detected in different parts of southern Africa (Terefe et al., 2014a; Pretorius et al., 2015). The genetic relatedness of isolates from the different countries indicates the possible dispersal of fungal spores across the region by wind and water or other mechanisms. Dispersal by wind is reportedly the main source of introduction of the fungus from southern Africa into Australia in the 1960s (Brown and Hovmøller, 2002; Kolmer, 2005; Allen-Sader et al., 2019; Visser et al., 2019; Corredor-Moreno and Saunders, 2020). Mechanical transfer by means of contaminated clothing or goods also plays a role in the spread of spores across borders, which explains the occurrence of new races in areas where they were not detected previously. Such new races often pose challenges because they may defeat known leaf rust resistance genes used in the region, leading to resistance breakdown. In order to cope with the continuous evolution of *Pt*, there is a need for regular and well-coordinated monitoring and surveillance of virulence changes in the countries across the region (Sharma-Poudyal et al., 2020). Essential information gathered through disease surveys can guide the deployment of appropriate conventional and cutting-edge breeding technologies to produce improved varieties or breeding lines with broad spectrum and durable resistance to existing and emerging *Pt* races.

Host–plant resistance has been shown to be a cost-effective and environmentally safe control strategy for rusts and other diseases (Johnson, 1981, 1984; Kolmer, 1996). One of the significant challenges is that *Pt* frequently acquires new and aggressive virulence that overcomes existing race-specific genes (Pretorius et al., 2007, 2015; Terefe et al., 2011; Boshoff et al., 2018). In southern Africa, the continued release of wheat cultivars with different race-specific resistance genes places intense selection pressure on *Pt* that could have led to the high diversity of rust populations, similar to other regions such as North America (Kolmer et al., 2007). For example, an increased frequency of virulence of newly identified leaf rust races against race-specific genes *Lr3*, *Lr15*, *Lr20*, and *Lr26* on commercial cultivars and breeding lines was reported in South Africa (Boshoff et al., 2018). However, host-plant resistance has considerable success where continuous surveys for *Pt* races and other rust diseases are integrated with pre-breeding, breeding, and post-release management efforts targeting rust resistance as demonstrated by the Borlaug Global Rust Initiative (BGRI) and associated regional and global projects (Evanega et al., 2014). To achieve a sustainable and cost-effective control strategy for leaf rust, a multidisciplinary and co-operative research approach involving many stakeholders including farmers, breeders, geneticists, pathologists, biotechnologists, and policymakers has to be in place. This strategy must include the exchange of resistant germplasm to safeguard border countries in the event of severe leaf rust outbreaks.

Germplasm exchange in southern Africa is aligned to the global wheat germplasm exchange network. The network consists of predominantly international public organizations such as the International Maize and Wheat Improvement Centre (CIMMYT)/Mexico and the International Centre for Agricultural Research in the Dry Areas (ICARDA)/Syria and several private multinational seed companies, each contributing to large germplasm pools. Some of the wheat genetic resources preserved in these centers are populations that were developed through dedicated pre-breeding and breeding programs targeting novel traits, including rust resistance. Wheat germplasm has been introduced across southern African borders between recipient countries, but the inability to meet seed import requirements has limited germplasm exchange. Further, policy issues on Intellectual Property ownership and benefit-sharing rendered limited germplasm exchange between African countries. These limitations may be circumvented through harmonized policy and regulations for enhanced germplasm exchange and through the use of recent advances in genetic and genomic technologies to deliver novel genetic resources with durable rust resistance.

In light of the above background, the objective of this review is to present the variability and distribution of *Pt* races in southern Africa. Focus is placed on describing the predominant races common across the region and the deployed leaf rust resistance genes in wheat. A comprehensive summary of effective leaf rust resistance genes available in different parts of the world that could be pyramided into local cultivars through advanced genetic and genomic technologies is also provided. Perspectives on the strides taken and milestones reached through collaborative research toward finding the best strategy to achieve durable leaf rust resistance in southern Africa and significant challenges encountered in the process are also discussed. Prospects of host induced gene silencing using the RNA interference (RNAi) technology has been highlighted as an option to enhance cultivar resistance against *Pt*.

## Genetic Variability and Distribution of *Pt* in Southern Africa

*Puccinia triticina* populations throughout the world are genetically diverse in terms of their molecular architecture as well as the combinations and complexity of their virulence patterns. This is influenced by the co-evolution of the pathogen with diverse wheats and genetic recombination of *Pt* races emerging from various *Triticum* species (Kolmer and Liu, 2000; Liu et al., 2014; Prasad et al., 2020). The genetic characteristics of all *Pt* populations is typical of diploid/dikaryotic populations, with a life cycle summarized by Bolton et al. (2008). The pathogen maintains high levels of variability through sequential mutations and sexual or asexual recombination, which lead to virulence shifts (Bolton et al., 2008). The importance and frequency of somatic recombination as a mechanism to induce variation in *Pt* races is yet unclear. Dispersal of rust races over large distances to new areas/regions where they were not detected before adds to the aforementioned sources of variability.

To understand the variability of *Pt* races in southern Africa, scheduled surveys and collections of leaf rust isolates are conducted annually based on protocols outlined by the Global Cereal Rust Monitoring System^1^. The involved institutions are the Agricultural Research Council-Small Grain (ARC-SG) and the University of Free State (UFS) in South Africa, Chitedze Research Station and CIMMYT in Malawi; the Crop Breeding Institute (CPB), Plant Protection Research Institute (PPRI), and Rattray Arnold Research Station, Seed Co., Ltd., in Zimbabwe; and Zambian Agriculture Research Institute, Mount Makulu Research Station, in Zambia. Results from the surveys are shared with the wheat breeding community through the global rust monitoring system developed by CIMMYT and other partners as part of the BGRI to mitigate the threat of wheat rust disease^2^.

### Tools for Detecting *Pt* Variability and New Race Introductions

#### Race Analysis

Traditional and genomic tools can be deployed to distinguish leaf rust physiologic races. Wheat differential varieties or lines form the basis of race analysis. Typically, near-isogenic lines (NILs) containing different leaf rust resistance genes/alleles are used to distinguish races by their phenotypic responses to different pathogen strains. Having NILs with single *Lr* genes in a uniform background of a highly susceptible cultivar or line facilitates research in understanding the genetics of host–parasite interactions (McCallum et al., 2016). The use of differential lines to distinguish physiological specialization of *P. triticina* was pioneered by Mains and Jackson (1926). Improvements were constantly made on the original leaf rust differential set through additions when new physiologic races were described. A series of NILs in a Thatcher background are now routinely used globally (Table 1). The original 12 sets of NILs was developed by Dr. Peter Dyck from the Cereal Research Centre, Agriculture and Agri-Food Canada in Winnipeg following work initiated by Dr. Robert G (Anderson, 1963; Long and Kolmer, 1989). Differential lines are grouped in sets of four. When the four lines are classified for resistance or susceptibility, there are 16 possible combinations. Additional sets are commonly used to supplement the standard differential lines (Huerta-Espino et al., 2011; Terefe et al., 2011). The North American nomenclature system for designating virulence combinations of *Pt* races is followed as a standard in most southern African countries as was proposed by the North American Wheat Leaf Rust Research Workers Committee (Kolmer et al., 2010). When 16 differential lines are used, for example, pathogenicity of a race is given a four-letter code, where the first three letters indicate the pathogenicity of the race on three sets of four lines. The fourth letter describes the pathogenicity of the race on the set used to supplement the standard 12 differential lines (Kolmer et al., 2010; McCallum et al., 2010). A number of sophisticated and modern tools can also be used to investigate variation in *Pt* populations including the use of DNA based markers and field pathogenomics which are described below.

**TABLE 1:** Common near isogeneic lines (NILs) in Thatcher background encompassing specific *Lr* genes [Adapted from McCallum et al. (2016)].

***Lr* gene**	**RL number**	**Pedigree**
*Lr1*^¥^	RL6003	Thatcher*6/Centenario
*Lr2a*^¥^	RL6016	Thatcher*6/Webster
*Lr2b*^¥^	RL6019	Thatcher*6/Carina
*Lr2c*^¥^	RL6047	Thatcher*6/Brevit
*Lr3a*^¥^	RL6002	Thatcher*6/Democrat
*Lr3bg*^¥^	RL6042	Bage/8*Thatcher
*Lr3ka*^¥^	RL6007	Thatcher*6/Klein Anniversario
*Lr9*	RL6010	Transfer (*Aegilops umbellulata*)/6*Thatcher
*Lr10*^¥^	RL6004	Thatcher*6/Exchange
*Lr11*^¥^	RL6053	Thatcher*6//E-1/Hussar
*Lr12*	RL6011	Exchange/6*Thatcher
*Lr13*	RL6001	Prelude*6/Loros
*Lr14a*^¥^	RL6013	Selkirk/6*Thatcher
*Lr14b*	RL6006	Thatcher*6/Maria Escobar
*Lr15*^¥^	RL6052	Thatcher*6/W1483
*Lr16*^¥^	RL6005	Thatcher*6/Exchange
*Lr17a*^¥^	RL6008	Klein Lucero/6*Thatcher
*Lr17b*^¥^	Harrier	Norin10/Brevor(seln14)//Kite sib/3/Kite
*Lr18*^¥^	RL6009	Thatcher*7/Africa 43
*Lr19*	RL6040	Thatcher*7/Translocation 4 (*Lr19* derived from *Agropyron elongatum*)
*Lr20*^¥^	RL6092	Thatcher*6/Timmo
*Lr21*	RL6043	Thatcher*6/RL5406(Tetra Canthatch/*Aegilops squarrosa* var *meyeri*-RL5289)
*Lr22a*	RL6044	Thatcher*7/RL5404(Tetra Canthatch/*Aegilops squarrosa* var *strangulata*-RL5271)
*Lr22b*	Thatcher	Marquis/Iumillo Durum//Marquis/Kanred
*Lr23*	RL6012	Lee 310/6*Thatcher
*Lr24*^¥^	RL6064	Thatcher*6/3/Agent//2*Prelude/8*Marquis
*Lr25*	RL6084	Thatcher*7/Transec
*Lr26*^¥^	RL6078	Thatcher*6/St-1-25
*Lr28*	RL6079	Thatcher*6/C-77-1
*Lr29*	RL6080	Thatcher*6//CS7D/Ag#11
*Lr30*^¥^	RL6049	Thatcher*6/Terenzio
*Lr32*	RL6086	Thatcher*6/3/Thatcher/*Aegilops squarrosa*//Mq(K)
*Lr33*	RL6057	Thatcher*6/PI58548
*Lr34*	RL6058	Thatcher*6/PI58548
*Lr35*	RL5711	Marquis-K*8//RL5344/RL5346 (*Triticum monococcum*)
*Lr37*	RL6081	Thatcher*8/VPM
*Lr38*	RL6097	Thatcher*6/T7Kohn
*Lr44*	RL6147	Thatcher*6/*Triticum speltoides* 7831 85GN 438
*Lr45*	RL6144	Thatcher*6/St-1
*Lr52*	RL6107	Thatcher*6/V336
*Lr60*	RL6172	Thatcher*3/V860
*Lr63*	RL6137	Thatcher*6/TMR5-J14-12-24
*Lr64*	RL6149	Thatcher*6/8404
*Lr67*	RL6077	Thatcher*6/PI250413

*^¥^Differential lines carrying these genes are used in southern Africa.*

#### Use of Molecular Markers for Race Diagnostics

DNA based molecular markers such as random amplified polymorphic DNA (RAPD) and amplified fragment length polymorphism (AFLP) have long been successfully used in mycology and plant pathology for the differentiation of species within the *Puccinia* genera (Kolmer et al., 1995; Liu and Kolmer, 1998). Kolmer and Liu (2000) distinguished groups of *P. triticina* isolates from international collections using RAPD markers. Results of sixty-nine isolates collected in Canada suggested that *Pt* populations in North America most likely evolved from several introductions that differed in molecular background (Kolmer, 2001). Co-dominant markers such as locus-specific microsatellites or simple sequence repeats (SSRs) and single nucleotide polymorphism (SNP) were later developed for *P. triticina* to distinguish heterozygote genotypes (Suenaga et al., 2003; Lagudah et al., 2006; Vida et al., 2009; Terracciano et al., 2013). A recent review by Kolmer et al. (2019) presents a detailed summary of the use of SSRs for characterization and identification of *Pt* isolates from different countries or continents.

In southern Africa, the use of DNA-based markers, particularly co-dominant markers such as SSRs and SNPs in race analysis and identification has been widely used to complement the traditional methodology of phenotyping (Visser et al., 2012; Pretorius et al., 2015; Boshoff et al., 2018), thus improving our understanding of the origin, phylogeny, and spread of *Pt* genotypes. A number of leaf rust races have been detected in various locations in southern Africa, including Malawi, Mozambique, South Africa, Swaziland, Zambia, and Zimbabwe (Table 2). Pretorius et al. (1987) detected races PDRS, SBDS, and SDDS in the early 1980s in South Africa. The earliest evidence of commonality in southern African *Pt* races was reported by Pretorius and Purchase (1990) from isolates collected in Zimbabwe, Zambia, and Malawi. Race SBDS, a race which was also detected in Zimbabwe in 1986, together with SCDS which was first detected in South Africa in 1988 (Pretorius and Purchase, 1990), were found to be similar except for the virulence of the latter to *Lr24* and *Lr26* genes. SBDS and SDDS were also found to have similar virulence/avirulence profiles, with SDDS being virulent to *Lr24*. Another race, SDDN was detected in the Western Cape Province of South Africa in 2005. This race was identical to SDDS, SCDS, and SFDS which were first detected in South Africa in 1987 (Pretorius and Le Roux, 1988), all sharing virulence to 11 *Lr* genes (Terefe et al., 2011).

**TABLE 2:** Avirulence/virulence profiles of frequent *Puccinia triticina* races detected in different localities in southern Africa from the 1980s^1^.

Race (NA notation)*	**Locality**	**Avirulence/virulence profile**	**References**
PDRS^2^	South Africa, Malawi	*Lr2a, 2b*, ***9***, *15*, ***16***, *17*, ***18***, *23, 26/1, 2c, 3a, 3bg, 3ka, 10, 11, 14a, 20, 24, 28, 30, B*	Pretorius and Le Roux, 1988; Pretorius and Purchase, 1990
SFDS	South Africa	*Lr3a, 3bg, 3ka*, ***9***, *11*, ***16***, ***18***, *26, 30/1, 2a, 2b, 2c, 10, 14a, 15, 17, 20, 23, 24, 28, B*	Pretorius and Le Roux, 1988
SBDS	South Africa, Zambia, Zimbabwe	*Lr3a, 3bg, 3ka*, ***9***, *11*, ***16***, ***18***, *24, 26, 20/1, 2a, 2b, 2c, 10, 14a, 15, 17, 20, 23, 28, B*	Pretorius et al., 1987
SDDS	South Africa	*Lr3a, 3bg, 3ka*, ***9***, *11*, ***16***, ***18***, *26, 30/1, 2a, 2b, 2c, 10, 14a, 15, 17, 20, 23, 24, 28, B*	Pretorius et al., 1987
SCDS	Malawi, South Africa	*3a, 3bg, 3ka*, ***9***, *11*, ***16***, ***18***, *24, 30/1, 2a, 2b, 2c, 10, 14a, 15, 17, 20, 23, 26, 28, B*	Pretorius et al., 1990; Terefe et al., 2014a
SDDN	South Africa	*Lr3a, 3bg, 3ka*, ***9***, *10, 11*, ***16***, ***18***, *26, 30/1, 2a, 2b, 2c, 14a, 15, 17, 20, 23, 24, 28, B*	Pretorius and Bender, 2010
CCPS	South Africa	*Lr1, 2a, 2b, 2c*, ***9*,** *11*, ***16***, ***18***, *20, 23, 24/3a, 3bg, 3ka, 10, 14a, 15, 17, 26, 28, 30, B*	Terefe et al., 2014a, b)
MCDS	Zimbabwe, Zambia, South Africa	*Lr2a, 2b, 2c, 3ka*, ***9***, *11*, ***16***, ***18***, *20, 24, 28, 30/1, 3a, 3bg, 10, 14a, 15, 17, 23, 26, B*	Terefe et al., 2014b
TCPS	Zimbabwe, Zambia	*Lr****9***, *11*, ***16***, ***18***, *24/1, 2a, 2c, 3a, 3ka, 10, 14a, 17, 26, 30, B*	
FBPT	Zimbabwe, South Africa	*Lr1, 2a, 2b*, ***9***, *11, 15*, ***16***, *20, 21, 23, 24, 26, 28/2c, 3a, 3ka, 10, 14a, 17, 18, 30, B*	Pretorius et al., 2015
CBPS	South Africa	*Lr1, 2a, 2b, 2c*, ***9***, *11, 12, 13, 15, 16, 18, 20, 24, 26, 27, 31/3a, 3bg, 3ka, 10, 14a, 17a, 30, B, Thatcher*	Boshoff et al., 2018
CFPS (3SA10)	South Africa	*Lr1, 2a, 2b, 2c*, ***9***, *11*, ***16***, ***18*,** *20/3a, 3bg, 3ka, 10, 14a, 15, 17a, 24, 26, 30, B, Thatcher*	Boshoff et al., 2018
CDPS	South Africa	*Lr1, 2a*, ***9***, *26, 11*, ***16***, ***18***, *26/3a, 3bg, 3ka, 10, 14a, 15, 17a, 20, 24, 30, B, Thatcher*	Boshoff et al., 2018
CFPS (3SA248)	South Africa	*Lr1, 2a*, ***9***, *2b, 2c, 11*, ***16***, ***18****/3a, 3bg, 3ka, 10, 14a, 15, 17a, 20, 24, 26, 30, B, Thatcher*	Boshoff et al., 2018

^1^This table is not necessarily exhaustive, only *P. triticina* races that have been predominant in southern Africa over the years are highlighted. Genes that are still effective against most races across regions are in bold. ^2^Letter codes are based on virulence pattern of the races on the original 12 differential lines plus a supplemental set (*LrB, Lr10, Lr14a, Lr18*). *NA = North America.

Using SSR markers, race PDRS was found to be genetically different from 10 other South African races, leading to speculation that it is a foreign introduction (Visser et al., 2012). Terefe et al. (2011) suggested that two new races CCPS and MCDS were found in South Africa as exotic introductions. The same study revealed that race CCPS shared 71 and 51% genetic similarities to the European races CCPSL and CCTSL, respectively. The CCPS race was even closer to MCPSS from the Czech Republic, validating the commonality of some races within regions. Race FBPT was detected in the Western Cape Province in 2010 (Terefe et al., 2014b). In a recent study, similar races (MCDS, TCPS, FBPT, and SCDS) have been detected by Pretorius et al. (2015) from 63 isolates obtained in different southern African countries during 2011–2013. MCDS and TCPS were found in Zimbabwe and Zambia whereas FBPT and SCDS were detected in Zimbabwe and Malawi, respectively. MCDS, FPBT, and SCDS were also detected in South Africa. The increased incidence of leaf rust disease from 2012 to 2016 in South Africa led to a detection of 4 new races (CBPS, CFPS, CDPS, and CFPS; Boshoff et al., 2018). CFPS (3SA10), CDPS (3SA38), and CFPS (3SA248) were found to be similar to each other, and only varied in virulence for *Lr20* and *Lr26* whereas CBPS (3SA115) was less virulent. Pretorius et al. (2007) revealed that the variation between most of the races identified in southern Africa was associated with virulence against the *Lr10, Lr14a*, *Lr17*, *Lr24*, and *Lr26* resistance genes.

Use of host plant differential lines remains key and complementary to the use of molecular technologies in identifying and distinguishing the different *Pt* races. Due to a sustained emergence of physiological races in the leaf rust pathogen, deploying differential host genotypes will have an added advantage to distinguishing resistance or susceptibility reactions of wheat genotypes to *Pt* races. When large sets of genotypes are screened for disease resistance, the phenotypic data can be associated with genomic data to identify putative and novel virulence and avirulence genes, as well as genes conferring resistance to particular races. The continuous use of race-specific resistance genes in breeding programs requires established surveillance systems of the leaf rust pathogen to scout for new races (Evanega et al., 2014).

#### Prospects of Field Pathogenomics

The technology that has not been explored to its fullest capacity for leaf rust race analysis is “field pathogenomics.” This is a relatively new method that uses the latest DNA sequencing technologies to generate high-resolution data that describe the diversity within a pathogen population directly from infected samples. A study by Hubbard et al. (2015) using field pathogenomics tools (RNAseq) successfully discovered a dramatic shift in a wheat yellow rust (*P. striiformis*) population in the United Kingdom, which can similarly be useful for leaf rust studies in southern Africa. With the increasing availability of fully sequenced pathogen genomes, this new high throughput technology will provide new insights into the biology, population structure and pathogenesis of pathogens of interest (Derevnina and Michelmore, 2015; Buen-Sancho et al., 2017) including rust. Detailed data on the genetic structure of *Pt* populations will provide new insights into the selective forces driving the evolution of new races. This will allow prediction of races that are a threat to specific wheat genotypes within southern Africa and provide an early-warning system for wheat genotype vulnerabilities and subsequent gene deployment providing spill-over benefits to wheat breeding programs in the region.

The *Pt* sequencing project was recently established forming part of the Fungal Genome Initiative at the Broad Institute, United States of America^3^. This initiative has provided the opportunity to generate expressed sequence tag (EST) libraries which have contributed to gene discoveries and stage-specific expression analysis (Xu et al., 2011). The generation of ESTs also has several applications including designing molecular markers such as SSRs, designing and constructing expression microarrays, genome annotation and comparative genomics to reveal relatedness between species. Based on information from a combination of EST sequences, *in silico* predictions and RNAseq transcript analysis, a high-quality reference genome sequence of *Pt* is now annotated and publicly available (Kiran et al., 2016). The availability of the annotated genome of the leaf rust pathogen as well as newly invented high-throughput DNA/RNA sequencing technologies will enable gene expression profiling of both the pathogen and the wheat plant to understand pathogenicity and plant resistance mechanisms during infection. Notably, Chandra et al. (2016) have made use of these newly available technologies to discover an array of wheat responses to leaf rust infection. It is foreseeable that this kind of work will be expanded to study all dynamics involved in the leaf rust-wheat pathosystem. Other questions which can be easily answered include the genetic response of the pathogen to fungicides and evolution.

Since shared virulence strategies may be used by different fungi to invade specific plant hosts, comparative analysis of the *Pt* genome with those of other pathogens may also offer a new and powerful approach to identify common and divergent virulence strategies as well as the evolutionary history of pathogen lineages. Gardiner et al. (2012) used comparative analysis to identify novel virulence genes in fungi infecting cereals. This reviewed the usefulness of comparative genomics in identifying potential pathogenicity factors in *Fusarium graminearium* and *Ustilago maydis*. The ever-increasing efficacy and decreasing costs of sequencing should motivate researchers, especially in southern Africa, to tap into the field of pathogenomics. This should shed more light on the potential origin, adaptation and interaction of southern African leaf rust races with the host, hence providing effective opportunities for leaf rust management.

## Current Control Strategies of Leaf Rust

Various options to control *Pt* including biological, cultural, chemical (fungicides) and host plant resistance are available. However, limited studies are conducted on the biological and cultural control options, leaving the last two options being widely used in southern Africa. Timely and accurate application of fungicides is effective in reducing both the incidence and severity of leaf rust in wheat. In the past years, regular fungicide spraying on commercial wheat fields of the Western Cape Province in South Africa contributed to the leaf rust fungus becoming less prevalent. Fungicides with active ingredients such as epoxiconazole, tebuconazole, pyraclostrobin, and trifloxystrobin remain effective for protecting yields of susceptible varieties from leaf rust attack. Nevertheless, fungicide use is not economically and environmentally sustainable, and can pose health hazards to people and animals, as well as phytotoxicity to the crop (Kolmer et al., 2007; Pretorius et al., 2007; McCallum et al., 2016). Further, repeated use of fungicides belonging to the same group may result in the development of fungicide resistant strains putting more pressure of formulating new fungicides that can combat new, normally more virulent mutant strains. Thus far, researchers are under pressure to ensure the reduction of overreliance on fungicides but at the same time promoting greater yield stability, subsequently generating a large proportion of returns on global economic investments in international wheat research (Chaves et al., 2013). Enhanced host plant resistance, and more importantly, the combination of several effective leaf rust resistance genes remains the most feasible, economic and environmentally friendly approach to ensure durable resistance (Ayliffe et al., 2008; McCallum et al., 2016). Wheat cultivars and breeding lines with multiple leaf rust resistant genes have significantly lower disease levels (German and Kolmer, 1992; Vanzetti et al., 2011; Tsilo et al., 2014), and the use of this kind of resistance has the potential to significantly reduce disease epidemics.

### Host Plant Resistance

Host plant resistance against *Pt* is grouped into two broad categories: (1) seedling resistance, which is conferred by single or major genes (often race-specific) and (2) adult plant resistance (APR) or partial or polygenic or slow rusting resistance caused by minor genes. APR is mainly race-non-specific, but it can also be race-specific and short-lived, being effective only against *Pt* isolates carrying the corresponding avirulence gene. Seedling resistance is expressed at all growth stages and is sometimes referred to as all-stage resistance (ASR). All-stage resistance is commonly qualitative and associated with a programed cell death defence response referred to as hypersensitive immunity. Adult plant resistance, on the other hand, is most effective in adult plants and is commonly quantitative. The mechanisms of action of seedling and APR genes, including their interaction with the pathogen avirulence (*Avr*) genes have been discussed in detail in previous reports (Johnson, 1984; Vanzetti et al., 2011; Evanega et al., 2014; Sekhwal et al., 2015). Silva et al. (2015) reported the progress made in the identification of QTL for leaf rust resistance. Durable resistance breeding programs should be guided by accurate information of the mechanism and impact of resistance conferred by target genes because it is often the mode of resistance and the right combination of resistance genes that determine the durability of resistance.

At present, more than 80 different leaf rust resistance genes and QTL spread throughout the A, B and D wheat genomes have been identified and cataloged (Kolmer, 2013; Sapkota et al., 2019). Most of these resistance genes and gene complexes were sourced from relatives of wheat such as *Thinopyrum elongatum* Zhuk. (*Lr 19*), *Aegilops tauschii* (*Lr21*), *Agropyron elongatum* (*Lr24*), *Secale cereale* L. (*Lr26*), *Aegilops peregrina* (*Lr59*) among others (McIntosh, 1975; Autrique et al., 1995; McIntosh et al., 1995; Marais and Botes, 2003). The transfer of these resistance genes from wild species to bread wheat has been successfully achieved globally. Breeding efforts have been undertaken to introgress various *Lr* genes into wheat breeding lines. A significant number of the *Lr* genes are race specific and generally conform to the “gene-for-gene” model proposed by Flor (1946), conferring resistance to pathogen races with corresponding *Avr* genes. They, therefore, lack durability, which was defined by Johnson (1984) as “the ability of a widely-deployed resistance gene to provide an economic level of protection over an extended period of time.” Hence major genes are frequently defeated by the appearance of new virulent races in the pathogen population through single-step mutations of the *Avr* gene and/or recombination or migration of new races (Samborski, 1985; Bolton et al., 2008).

Most cultivars or breeding lines that were developed earlier in southern Africa are no longer used since they largely carried race-specific genes such as *Lr1, Lr3a, Lr10, Lr13, Lr14a, Lr17b, Lr24, Lr26, Lr27*, and *Lr31*, which were defeated by new virulent races. For example, a South African wheat cultivar (SST44) carrying *Lr24* was widely used in the 1980’s and the prevalence of new virulent races substantially increased vulnerability of the cultivar to leaf rust. Other varieties including Gouritz, Dipka, Flamink, SST101, and Zaragoza which were grown abundantly in South Africa were reportedly susceptible (Pretorius et al., 2007). In some cultivars, however, the combination of some of these leaf rust resistance genes remains effective owing to the absence of specific virulence combinations in local races (Pretorius et al., 1996). Maintenance of these race-specific gene combinations is not sustainable in breeding programs due to a risk of creating races of *Pt* isolates with multiple-gene resistance, leading to hypervirulent pathogen populations. Constant identification and selection of new sources of resistance therefore remain important in most rust resistance breeding programs. The identification of new sources with effective and durable resistance genes allows for the efficient incorporation of these target genes into germplasm pools (Kolmer, 1996). In this case, local cultivars can be improved with genes and QTLs outsourced from lines showing durable leaf rust resistance from other countries. For instance, the Brazilian cultivar, Toropi, exhibited durable APR since its release in 1965 (Casassola et al., 2015), suggesting its value in resistance breeding programs. Kolmer et al. (2018a) identified that the APR resistance in the Brazilian cultivar was conditioned by *Lr78*, mapping on chromosome 5DS and three other minor QTL. As a result, this cultivar was introduced to South Africa and is currently being used in resistance breeding at ARC-Small Grain. Additional APR genes including *Lr74*, *Lr75*, and *Lr77* have been identified and mapped to chromosomes 3BS, 1BS, and 5DS, respectively (Singla et al., 2017; Kolmer et al., 2018b, c).

Resistance conferred by APR genes is commonly undetectable at a seedling stage but is often effective against a wide range of known *Pt* physiologic races. APR genes confer partial resistance, regulating the pathogen’s effectiveness by producing fewer and smaller uredinia which are surrounded by chlorosis. APR often provides long-term and durable resistance. *Lr34*/*Sr57*/*Yr18/Pm38* and *Lr67/Sr55/Yr46/Pm46* are some of the few cloned and sequenced adult plant (race-non-specific) resistance genes (Lagudah et al., 2006; Moore et al., 2015) in plants conferring durable resistance to leaf rust, stripe rust, stem rust, powdery mildew, and barley yellow dwarf virus (Singh, 1993). Adult plant resistance genes may have small to intermediate effects when used individually, while high levels of resistance are achieved by combining four to five genes with additive effects (German and Kolmer, 1992; Kloppers and Pretorius, 1997; Singh et al., 2000; Vanzetti et al., 2011; Tsilo et al., 2014). Other adult plant (race-non-specific) resistance genes, including *Lr46/Yr29/Pm39*, *Sr2/Yr30*, and *Lr68* have been characterized (Singh et al., 1998; Hiebert et al., 2010; Herrera-Foessel et al., 2012). Table 3 summarizes some of the effective seedling and APR genes that have been deployed in most breeding programs in southern Africa and around the world. Availability of DNA markers for these genes makes it easier to deploy host–plant resistance effectively, even when race phenotyping is not possible to conduct gene postulations.

**TABLE 3:** Chromosome location, description, linked DNA markers and references for leaf rust resistance genes still deployed against most *Puccinia triticina* races in southern Africa and around the world.

***Lr* gene**	**Chromosomal region**	**General description**	**Linked DNA marker**	**Original source**	**References**
***Major Genes***					
*Lr9*	6BL	Confers resistance to leaf rust	*cMWG684* *PSR546* *J13*	*Aegilops umbellulata*	Schachermayr et al., 1994
*Lr19*	7DL	Effective against all races of leaf rust in South Africa	*STSLr19_130_*	*Agropyron elongatum*	Prins et al., 2001
*Lr37*	2AS	Confers resistance to leaf rust	*Xcmwg682*	*Triticum ventricosum*	Helguera et al., 2003
*Lr39*	1DS	Allelic or identical to *Lr21*		*Triticum tauschii*	
*Lr41*	1D	Confers resistance to leaf rust	*BARC124* *GMM210* *GDM35*	*Triticum tauschii*	Sun et al., 2009
***Minor Genes***					
*Lr27/Yr30/Sr2/Pbc1*	3BS	Associated with pseudo-black chaff (morphological marker); confers slow rusting resistance to leaf rust, yellow rust, stem rust and powdery mildew	*csSr2-SNP*	*Triticum turgidum*	Mago et al., 2011; McNeil et al., 2008
*Lr34/Yr18/Sr57/Pm38/Ltn1*	7DS	Confers slow rusting resistance to leaf rust, yellow rust, stem rust, powdery mildew and yellow dwarf virus Associated with leaf tip necrosis (morphological marker) Cloned	*csLV34* *Swm10* *cssfr1 to cssfr7*	*Triticum aestivum*	Lagudah et al., 2006
*Lr46/Yr29/Sr58/Pm39/Ltn2*	1BL	Confers slow rusting resistance to leaf rust and stripe rust	*csLV46* *PstAAGMseCTA-1*	*Triticum aestivum*	William et al., 2003
*Lr67/Yr46/Sr55/Pm46/Ltn3*	4DL	Confers slow rusting resistance to leaf rust and stripe rust Cloned Associated with leaf tip necrosis (morphological marker)	*gwm165*	*Triticum aestivum*	Moore et al., 2015
*Lr68*	7BL	Confers slow rusting resistance to leaf rust	*Psy1-1* *gwm146* *cs7BLNLRR* *scGS*	*Triticum aestivum*	Herrera-Foessel et al., 2012
*Lr74*	3BS	Confers resistance to leaf rust	*cfb5006*	*Triticum aestivum*	Kolmer et al., 2018a
*Lr75*	1BS	Confers resistance to leaf rust	*gwm604* *swm271*	*Triticum aestivum*	Singla et al., 2017
*Lr77*	3BL	Confers resistance to leaf rust	*IWB10344*	*Triticum aestivum*	Kolmer et al., 2018b
*Lr78*	5DS	Confers resistance to leaf rust	*IWA6289*	*Triticum aestivum*	Kolmer et al., 2018c

*The most useful diagnostic markers are underlined.*

### Durable Leaf Rust Resistance

Resistance is considered durable if it remains effective within a cultivar under cultivation for a significant number of years over a substantial area with favorable conditions for the respective pathogen (Johnson, 1981; Johnson, 1984). Several studies have demonstrated that durability of leaf rust resistance is more likely to be of adult plant type than of seedling type and is often linked to genes or gene loci that confer durable resistance to other rusts and diseases, as in the case of *Lr34/Yr18/Sr57* (Singh, 1992), *Lr46/Yr29/Pm39* (Kolmer et al., 2015), *Lr67/Yr46* (Herrera-Foessel et al., 2014); and probably *Lr68* (Herrera-Foessel et al., 2011), *Lr74* (Kolmer et al., 2018b) *Lr75* (Singla et al., 2017), *Lr77* (Kolmer et al., 2018c), *Lr78* (Kolmer et al., 2018a) and though the effect of the latter on other diseases has not been reported yet. However, successful integration or pyramiding of a number of single *Lr* genes conferring complete resistance with a few minor genes or APR genes could result in a significantly broader durable resistance over a significant number of years, a strategy advocated by BGRI. When successfully combined, resistance genes often complement each other, giving reactions different from those given individually (Kloppers and Pretorius, 1997). Furthermore, the effects of simultaneous mutations in the pathogen on virulence against multiple resistance genes is greatly reduced as compared to single genes, therefore, resistance remains effective for longer (Schaefer et al., 1963).

Kloppers and Pretorius (1997) observed active complementation of *Lr34* (race-non-specific) and *Lr13* (race-specific) with both genes improving resistance on the selected lines even with the presence of races possessing virulence for the *Lr13* gene. German and Kolmer (1992) and Kolmer et al. (2007) also reported prolonged and positive interaction of resistance genes *Lr16*, *Lr23*, and *Lr34* in North America. The complementary effect of combining resistance genes was also tested by Pretorius et al. (1996) transferring *Lr21*, *Lr29*, *Lr32*, *Lr34*, *Lr35*, *Lr36*, *Lr37*, *Lr41*, and *Lr42* to South African cv. Palmiet and Karee through the backcrossing method. Likewise, Vanzetti et al. (2011) found that seedling resistance genes such as *Lr16, Lr47, Lr19, Lr41, Lr21, Lr25*, and *Lr29* provided durable resistance when combined with APR genes such as *Lr34, Sr2*, and *Lr46* in Argentina. A recent study by Silva et al. (2015) demonstrated the relevance of combining *Lr34*, *Lr68*, and *Sr2* to increase leaf rust resistance, showing the importance of using APRs as key genes to be deployed in breeding programs to attain high levels of resistance. Combinations of partial resistance genes in the CIMMYT breeding program have conferred adequate resistance to leaf rust in the field for several years (Singh et al., 2005). Breeding lines from this program are widely used in southern Africa. Local researchers should propose breeding strategies and efficient use of tools to assist in outsourcing and introgressing new sources of rust resistance into suitable backgrounds. Efficient screening of a large number of breeding lines and varieties to determine the presence and frequency of resistance alleles in breeding programs is also necessary. The epistatic genetic effects of race-specific genes and the partial resistance response of APR genes complicates breeding strategies aimed at recombination of these genes in wheat cultivars. This necessitates the development of molecular markers to assist in gene pyramiding. However, the limited availability of diagnostic molecular markers significantly impedes the screening and gene pyramiding processes. Pre-breeding efforts are well undertaken in many institutions around the world to address the challenges. In South Africa, the University of the Free State, Stellenbosch University and ARC amongst others have invested significantly in wheat leaf rust research and resistance breeding. Some of the effective genes available include *Lr9, Lr19, Lr29, Lr34, Lr45, Lr47, Lr51*, and *Lr52*.

The development of more precise and affordable molecular tools like Kompetitive Allele Specific PCR (KASP) markers, the use of next generation sequencing (NGS) technologies and QTL mapping have proved crucial in identifying more APR genes and other effective race specific genes that can be combined to achieve stable and durable resistance. Several studies supporting the use of these tools, looking at their advantages and occurring gaps are available. A recent NGS based transcript analysis by Satapathy et al. (2014), which revealed the differential responsiveness of pathogen defence related to WRKY genes, with the potential of detecting leaf rust specific resistance genes encourages utilization of advanced technologies in rust breeding programs. Further, Li et al. (2014) provides a comprehensive summary of the genomic resources for durable leaf rust resistance breeding including eighty previously reported QTLs, and give prospects of fine mapping and cloning of these QTLs due to advances in NGS technologies. Therefore, southern African countries which have not yet invested in some of these advanced genomic tools can equally benefit from other partners through regional and global collaborations, improving their capacity to develop cultivars with durable resistance. However, local efforts to develop tools for resistance breeding, studying resistance mechanisms and pathogen genetic diversity must be intensified.

## Challenges in Breeding for Durable Leaf Rust Resistance in Southern Africa

As much as rust pathogens, in general, have great economic importance, limited information is available on the genes and factors required for pathogenesis and virulence. Adding to this, accurate information on the effect of the environment and full knowledge of the identity of effective resistance genes in the host, their mode of action when interacting with the *Avr* gene, and the interaction of slow rusting resistance genes with other resistance genes or gene loci to achieve stable and durable rust resistance across environments is still limited. It has been reported in several studies that temperature variations play a significant role in the expression of many resistance genes. A study by Herrera-Foessel et al. (2011) found that in Ciudad Obregon, Mexico, the effect of *Lr68* was smaller than the effect of *Lr34, Lr67*, or *Lr46* in the 2007–2008 and 2008–2009 seasons, while *Lr68* showed stronger effects than *Lr46* in the 2010–2011 season in the same studied environment. The two cropping seasons varied greatly in temperature as the 2010–2011 season proved to be significantly cooler than the previous seasons, suggesting that *Lr68* may be expressed better at lower temperatures. Studies by Pretorius et al. (1984) and Kolmer (1996) also highlighted the effects of temperature on the expression of the APR gene *Lr13*. In the former study, *Lr13* was expressed at 25°C, resulting in an avirulent infection type in seedling plants exposed to three isolates of *Pt* from Mexico, China and Chile. Kolmer (1996) on the other hand showed that *Pt* isolates from North America have high infection types to seedlings with *Lr13* regardless of temperature, but many of the same isolates have low infection types to adult plants with *Lr13*.

Limited public funding for sustained research on both the host as well as the pathogen and its variability, created a bottleneck in breeding for durable leaf rust resistance in most southern African countries. For example, despite the contribution of the ARC in improving the performance of the agricultural sector in South Africa, public funding (through Parliamentary Grant) to the ARC has been declining in real terms over the recent years (Dlamini et al., 2015). This impacts the ability of the ARC to effectively implement and support its various research programs, including wheat breeding programs. Hence, an investment strategy that supports sustained research programs geared toward wheat varietal improvement as well as identifying and addressing the ever-evolving rust races is necessary (Nhemachena et al., 2019). Plant breeders with the help of agricultural economists, need to estimate the benefits derivable from wheat varietal improvement research including breeding for durable rust resistance, to provide important arguments to decision-makers in the prioritization and allocation of public funding to wheat varietal research and other research needs. The training of future plant breeders, pathologists as well as bioinformaticians in collaboration with universities in Africa, will also generate immeasurable benefits in maintaining or conserving valuable genetic materials for wheat and other crops, and generate varieties that integrate specific public-good traits such as durable rust resistance.

## The Future of Durable Resistance Breeding

Much effort is still required than just relying on the phenotypic and genetic characterization of individual resistance genes to achieve durable resistance. A more system-oriented approach is needed, and this may include developing durable transgenic hosts and silencing of essential genes in the pathogen by expressing small interfering RNAs in the host (HIGS). Modest progress has been made in engineering durable resistance to wheat rust, especially leaf rust which causes considerable damage to wheat production. Resistance genes are continually being characterized and mapped in wheat and its relatives with only a few durable leaf rust resistance genes mapped. Adding to this, map-based cloning in wheat has become easier with the availability of whole genome sequencing. However, limited work has been done in cloning multiple effective leaf rust resistance genes. Some of the cloned leaf rust resistance genes include *Lr1* (Cloutier et al., 2007), *Lr10* (Feuillet et al., 2003), *Lr21* (Huang et al., 2003), *Lr34* (Lagudah et al., 2006), and *Lr67* (Moore et al., 2015). Cloning more effective resistance genes is needed for incorporation into resistance gene cassettes which could be successful in breeding for durable rust resistance. The advantage of cassettes is that the genes segregate as a unit. The genome editing technology can also prove useful for both the host and the pathogen, aiding in sequentially inserting multiple cassettes or incorporating multiple genes including non-host resistance genes at a single target site in the plant genome.

### Host Induced Gene Silencing

The discovery of RNAi technology has provided new opportunities to explore engineering of resistance against some biotrophic pathogens in plants by inducing silencing of genes essential for pathogen virulence. RNAi is an intrinsic cellular mechanism shared by all multicellular eukaryotes with apparent roles in gene regulation and defence against viral infection (Baulcombe, 2004). The mechanism occurs post-transcriptionally and converts double stranded (ds) RNA into short RNA duplexes of 21–28 nucleotides in length, followed by the guided cleavage of complementary mRNA by the generated sequence-specific single-stranded RNAs termed short interfering (si) RNAs (Watson et al., 2005; Small, 2007). The RNAi pathway can also be activated by viral RNAs and is a major line of defence against RNA viruses. Virus-induced gene silencing (VIGS) uses viral vectors to produce dsRNA of a target gene fragment and triggers RNAi silencing. The mechanism has been exploited extensively and has become a powerful functional genomic tool to silence any gene of interest by introducing target gene sequences into cells or organisms. Recent studies have demonstrated that the expression of silencing constructs in plants designed on fungal genes can silence the expression of their target respective genes in invading pathogenic fungi (Nunes and Dean, 2012). The barley stripe mosaic virus (BSMV) is popularly used as a vector in wheat to scrutinize candidate genes for their involvement in rust resistance. Yin et al. (2011) made use of BSMV to express dsRNA from *Puccinia* genes in plants to determine whether silencing can be delivered to the pathogen and suppress the expression of the fungal genes. Results from this study clearly showed that some *P. striiformis* genes can be silenced through a host-induced RNAi system. This may yet prove to be a viable technology to analyse gene functions in rust fungi and control wheat rust disease in southern Africa.

## Conclusion and Future Prospects

The variability and constant evolution of wheat leaf rust populations exhort huge pressure on wheat breeders and researchers in general to be constantly vigilant against the emergence of new rust races. This requires timeous monitoring and collaborative surveillance of changes in the virulence patterns among rust pathogens in each country and across regions. Durable host plant resistance to leaf rust is one of the most critical traits that breeding programs should invest in, permitting a reduction in the use of fungicides as well as promoting greater stability and sustainability of yield levels. The use of highly sophisticated and high-throughput tools such as field pathogenomics, transgenics, genome editing, and NGS to study both the host and the pathogen will assist in ultimately achieving broad spectrum and durable leaf rust resistance in wheat. This will subsequently result in a realization of large proportions of returns on global economic investments in international wheat research. A multidisciplinary approach involving pathologists, breeders, geneticists, physiologists, agronomists and bioinformaticians at different stages of research and development is necessary to develop an improved cultivar with stable and durable leaf rust resistance through host plant resistance approach. The extensive training of young researchers specifically in the field of plant–pathogen interaction and in the use of high-throughput technologies mentioned above, in collaboration with universities in Africa, will also generate immeasurable benefits in maintaining or conserving valuable genetic materials for wheat and other economically important crops and generate varieties that integrate specific public-good traits such as durable rust resistance.

## Author Contributions

SF wrote the first draft of the article and finalized with contributions by KN, LM, TT, TJT, and HS.

## Conflict of Interest

The authors declare that the research was conducted in the absence of any commercial or financial relationships that could be construed as a potential conflict of interest.

## Footnotes

1 https://rusttracker.cimmyt.org/

2 www.globalrust.org/

3 www.broad.mit.edu/annotation/genome/puccinia_group/MultiHome.html/
